# Pim-2 kinase inhibits inflammation by suppressing the mTORC1 pathway in atherosclerosis

**DOI:** 10.18632/aging.203547

**Published:** 2021-09-21

**Authors:** Minqi Liao, Feng Hu, Zhiqiang Qiu, Juan Li, Chahua Huang, Yan Xu, Xiaoshu Cheng

**Affiliations:** 1 The Department of Cardiovascular Medicine, The Affiliated Dongguan Hospital of Southern Medical University, Dongguan, Guangdong, China; 2 The Department of Cardiovascular Medicine, The Second Affiliated Hospital of Nanchang University, Nanchang, Jiangxi, China; 3 The Department of Orthopedics, The Second Affiliated Hospital of Nanchang University, Nanchang, Jiangxi, China; 4 The College of Pharmacy, Nanchang University, Nanchang, Jiangxi, China

**Keywords:** Pim-2, atherosclerosis, inflammatory, mTORC1

## Abstract

Background: Inflammatory immunity theory has raised considerable concern in the pathogenesis of atherosclerosis. Proviral integration site of murine 2 (Pim-2) kinases functions in apoptosis pathways and the anti-inflammatory response. Here, we investigated whether Pim-2 kinase inhibits atherosclerotic inflammation by suppressing the mTORC1 pathway.

Methods: An atherosclerosis animal model was established by feeding ApoE ^-/-^ mice a high-fat diet. THP-1-derived macrophages were subjected to ox-LDL (50 μg/ml, 24h) conditions *in vitro* to mimic the *in vivo* conditions.

Result: The protein expression of Pim-2 was upregulated in ox-LDL-treated THP-1-derived macrophages and an atherosclerotic mouse model. Additionally, ox-LDL upregulated the protein expression of p-mTOR, p-S6K1 and p-4EBP1, intracellular lipid droplets, free cholesterol and cholesterylester and the mRNA expression of inflammatory cytokines, including IL-6, MCP-1, TLR-4 and TNF-α, in THP-1-derived macrophages. Functionally, overexpressed Pim-2 (Pim-2 OE) attenuated atherosclerotic inflammation associated with the mTORC1 signaling pathway *in vitro* and *in vivo*, whereas knocked down Pim-2 (Pim-2 KD) markedly promoted atherosclerotic inflammation associated with upregulation of the mTORC1 signaling pathway. The plaque areas and lesions in the whole aorta and aortic root sections were alleviated in ApoE ^-/-^ mice with Pim-2 OE, but aggravated by Pim-2 KD. Additionally, an mTOR agonist (MHY1485) counteracted the anti-inflammatory effect of Pim-2 in ox-LDL-treated THP-1-derived macrophages after Pim-2 OE, whereas rapamycin rescued atherosclerotic inflammation in ox-LDL-treated THP-1-derived macrophages after Pim-2 KD. Furthermore, si-mTOR and si-Raptor alleviated the atherosclerotic proinflammatory effect in ox-LDL-treated THP-1-derived macrophages in a the background of Pim-2 KD.

Conclusions:These results indicated that Pim-2 kinase inhibits atherosclerotic inflammation by suppressing the mTORC1 pathway.

## INTRODUCTION

The mortality of cardiovascular and cerebrovascular disease is increasing yearly and atherosclerosis is the main cause of this ischemic disease [1]. Inflammatory immunity plays an important role in initiating atherogenesis and eventually destabilizing atherosclerotic lesions [2–6]. However, the exact molecular mechanism of atherosclerotic inflammation remains to be fully elucidated.

Mammalian target of rapamycin (mTOR) is a conserved serine/threonine protein kinase that exists in the cytoplasm in a stable state [7]. When activated by insulin growth factor, inflammation and other signals, it enters the nucleus to regulate downstream target molecules [8]. The mTOR signaling pathway senses and integrates various environmental cues to regulate organismal growth and homeostasis [9]. mTORC1 is a complex formed by the binding of mTOR protein with Raptor and works through phosphorylated 4EBP1 and S6K1, whereas mTORC2 is a complex formed by the binding of mTOR protein and Rictor [9]. Clinical studies have shown that scaffolds containing rapamycin-coated immunosuppressants prevent AS formation and restenosis [10, 11]. Ding et al. [12] found that atherosclerotic plaques were significantly reduced in LDLR^-/-^ mice fed a western-type diet transplanted with bone marrow from macrophage-Raptor knockout mice, and the macrophage contents and expression of inflammatory factors in atherosclerotic plaques were significantly reduced. Ma et al. [13] found that inflammation increased the accumulation of lipids in the aorta of ApoE ^-/-^ mice and vascular smooth muscle cells, which depended on the increased activity of mTORC1.

Proviral integration sites of murine (Pim) kinases are a distinct class of serine/threonine-specific kinases comprising Pim-1, Pim-2 and Pim-3. Studies have shown that Pim kinase plays an important role in the cell cycle and apoptosis by interacting with apoptotic genes or phosphorylating different substrates [14, 15]. Previously, we found that Pim-2 protects H9c2 cardiomyocytes from hypoxia/reoxygenation-induced apoptosis by downregulating Bim expression [16]. Interestingly, Pim-2 also has an anti-inflammatory effect [17, 18]. Geng et al. [17] found that the inflammatory response of rheumatoid arthritis was attenuated by activating the Pim2/mTORC1 pathway. Pim-2 slows down the development of asthma by inhibiting the secretion of inflammatory factors in Tregs [18].

Considering that the exact role of Pim-2 in atherosclerosis remains unknown, we investigated whether Pim-2 kinase inhibits atherosclerotic inflammation by suppressing the mTORC1 pathway in ox-LDL-treated THP-1-derived macrophages and ApoE ^-/-^ mice fed a high-fat diet.

## RESULTS

### Ox-LDL upregulates the expression of Pim-2, the mTOR pathway and inflammatory cytokines in THP-1-derived macrophages

**Figure 1:**
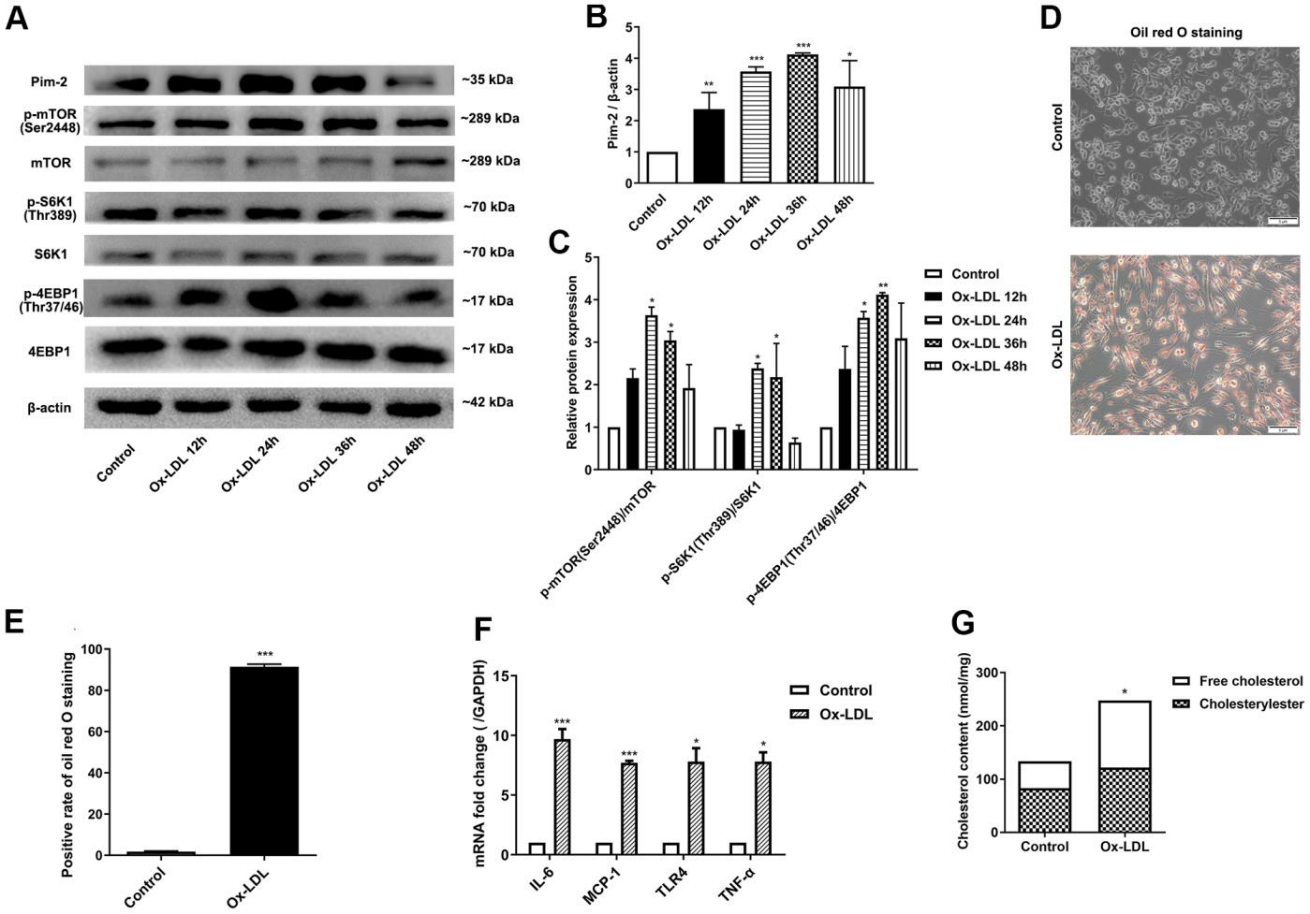
**Ox-LDL upregulates the expression of Pim-2, the mTOR pathway and inflammatory cytokines in THP-1-derived macrophages.** THP-1-derived macrophages were sequentially treated with 50 μg/ml of ox-LDL for 12 h, 24 h, 36 h, and 48 h. (**A**) Representative western blot analysis of Pim-2, p-mTOR (Ser2448) and mTOR, p-S6K1 (Thr389) and S6K1, and p-4EBP1 (Thr37/46) and 4EBP1. β-Actin was used as a loading control. (**B**, **C**) Corresponding densitometric analysis of blots in (**A**). (**D**) Intracellular lipid droplets were stained with oil red O working solution in THP-1-derived macrophages treated with 50 μg/ml of ox-LDL for 24 h. (**E**) Quantification of positive staining for lipid droplets (n=3 per group). (**F**) mRNA expression of inflammatory cytokines, including IL-6, MCP-1, TLR-4 and TNF-α, was determined by quantitative RT-PCR in ox-LDL-treated THP-1-derived macrophages; values normalized to the housekeeping gene GAPDH. (**G**) The concentrations of TC, FC, and CE were determined using the TC/FC Quantification Assay (n=3 per group). The data are represented as the means ± SD of three independent experiments; *P < 0.05, **P < 0.01, ***P < 0.001 versus the control group.

To determine whether ox-LDL affects the expression of Pim-2 and mTOR signaling pathway, THP-1-derived macrophages were sequentially treated with 50 μg/ml of ox-LDL for 12 h, 24 h, 36 h, and 48 h, respectively. Next, total protein was extracted and processed for western blot analysis. The protein levels of Pim-2 increased gradually and reached a peak at 36 h in response to ox-LDL stimulation (Figure 1A, 1B). Similarly, the protein expression levels of p-mTOR(Ser2448), p-S6K1(Thr389), and p-4EBP1(Thr37/46) increased gradually and reached a peak at 24 h or 36 h in response to ox-LDL stimulation (Figure 1A, 1C). Therefore, 24 h was selected as a suitable time point to investigate the role of Pim-2 and mTOR signaling pathways in atherosclerosis in subsequent experiments.

Intracellular lipid droplets were stained with oil red O working solution. Lipid droplets were significantly increased in ox-LDL-treated THP-1-derived macrophages (Figure 1D, 1E). Compared with the control group, the concentrations of TC, FC, and CE were significantly increased in ox-LDL-treated THP-1-derived macrophages (133.4 ±1.7 nmol/mg versus 247.3 ±26.6 nmol/mg (p<0.05), 83.2±12.0 nmol/mg versus 121.8 ±17.6 nmol/mg(p<0.05), 121.8 ±17.6 nmol/mg versus 125.6 ±11.4 nmol/mg (p<0.01), respectively; Figure 1G). Moreover, the mRNA expression of inflammatory cytokines, including IL-6, MCP-1, TLR-4 and TNF-α, was significantly increased in ox-LDL-treated THP-1-derived macrophages (Figure 1F).

### Overexpression of Pim-2 attenuates the inflammatory response in THP-1-derived macrophages

**Figure 2:**
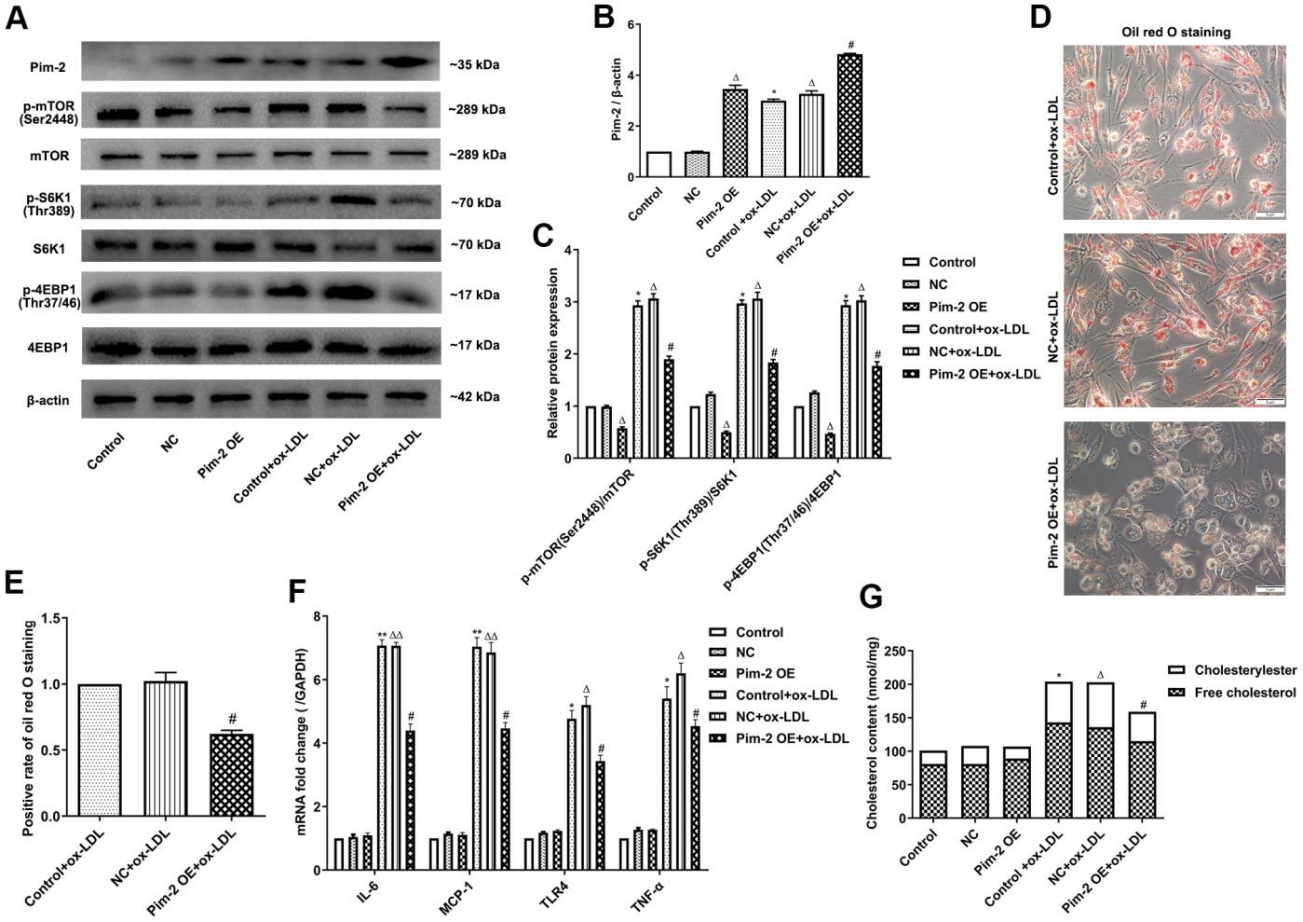
**Overexpression of Pim-2 attenuates the inflammatory response in THP-1-derived macrophages.** (**A**) Representative western blot analysis of Pim-2, p-mTOR (Ser2448) and mTOR, p-S6K1 (Thr389) and S6K1, and p-4EBP1 (Thr37/46) and 4EBP1 in ox-LDL-treated THP-1-derived macrophages after Pim-2 OE. β-Actin was used as a loading control. (**B**, **C**) Corresponding densitometric analysis of blots in (**A**). (**D**) Intracellular lipid droplets were stained with oil red O working solution in ox-LDL-treated THP-1-derived macrophages after Pim-2 OE. (**E**) Quantification of positive staining for lipid droplets (n=3 per group). (**F**) mRNA expression of inflammatory cytokines, including IL-6, MCP-1, TLR-4 and TNF-α, was determined by quantitative RT-PCR in ox-LDL-treated THP-1-derived macrophages after Pim-2 OE; the values were normalized to the housekeeping gene GAPDH. (**G**) The concentrations of TC, FC, and CE were determined using the TC/FC Quantification Assay (n=3 per group). The data are represented as the means ± SD of three independent experiments; *P < 0.05, **P < 0.01, versus the control group; ^Δ^*P* < 0.05, ^ΔΔ^*P* < 0.01, versus the NC group; ^#^*P* < 0.05 versus the ox-LDL-treated NC group.

Compared with the control group, the protein expression levels of p-mTOR(Ser2448), p-S6K1(Thr389), and p-4EBP1(Thr37/46) were significantly elevated in ox-LDL-treated THP-1-derived macrophages, but markedly decreased after overexpression of Pim-2 (Pim-2 OE) (Figure 2A–2C). As expected, compared with the ox-LDL-treated negative control group (NC group), intracellular lipid droplets were significantly decreased in ox-LDL-treated THP-1-derived macrophages after Pim-2 OE (Figure 2D, 2E). Compared with the ox-LDL-treated NC group, the concentrations of TC, FC, and CE were also markedly reduced in ox-LDL-treated THP-1-derived macrophages after Pim-2 OE (201.5 ±15.2 nmol/mg versus 157.6 ±11.3 nmol/mg (p<0.05), 135.8±10.7 nmol/mg versus 114.8 ±10.1 nmol/mg (p<0.05), 66.7 ±12.3 nmol/mg versus 43.8 ±10.9 nmol/mg (p<0.05), respectively; Figure 2G). Additionally, compared with the control group, the mRNA expression of inflammatory cytokines was significantly increased in ox-LDL-treated THP-1-derived macrophages, but markedly decreased after Pim-2 OE (Figure 2F).

### Silencing of Pim-2 promotes the inflammatory response in THP-1-derived macrophages

**Figure 3:**
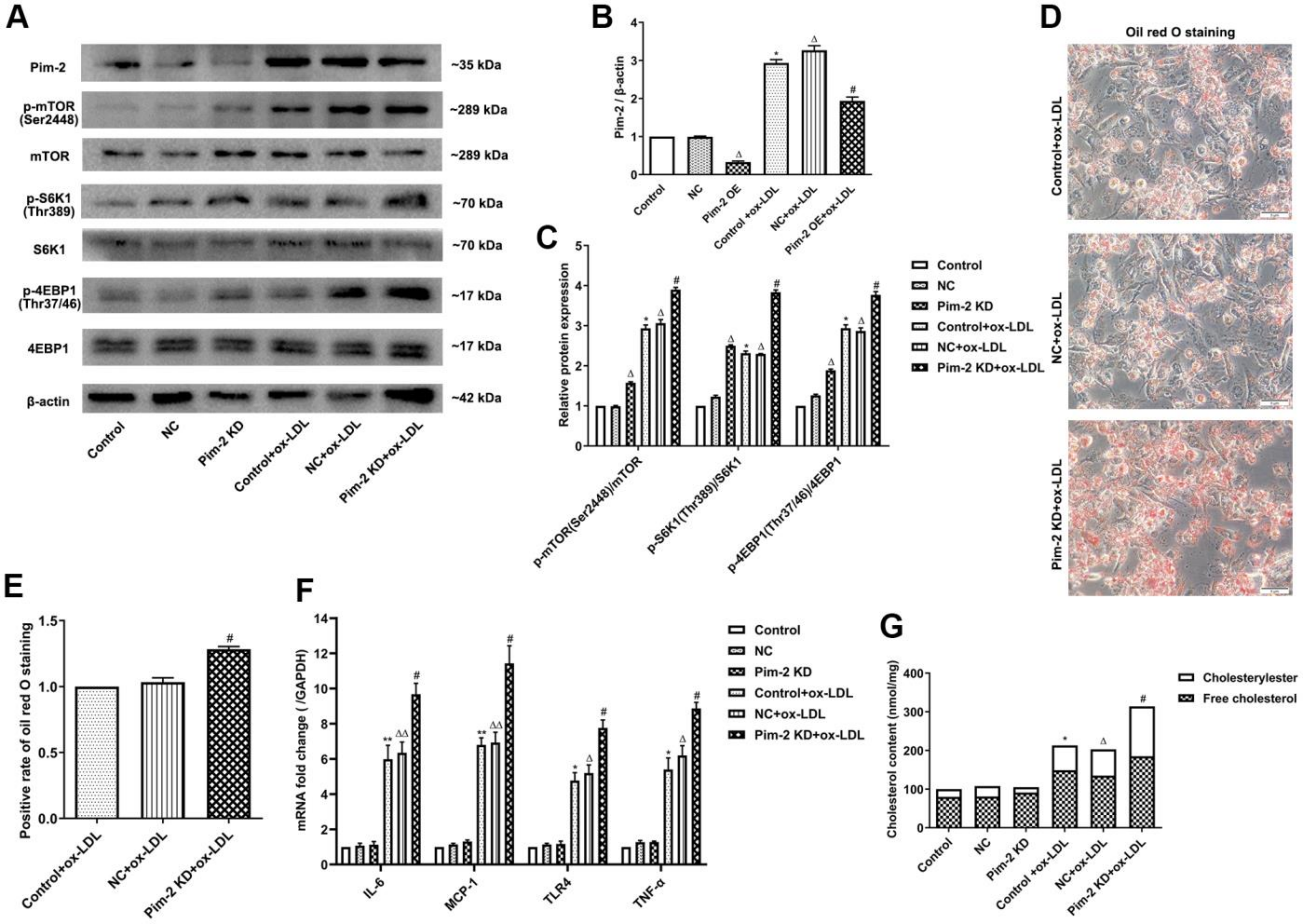
**Silencing of Pim-2 promotes the inflammatory response in THP-1-derived macrophages.** (**A**) Representative western blot analysis of Pim-2, p-mTOR (Ser2448) and mTOR, p-S6K1 (Thr389) and S6K1, and p-4EBP1 (Thr37/46) and 4EBP1 in ox-LDL-treated THP-1-derived macrophages after Pim-2 KD. β-Actin was used as a loading control. (**B**, **C**) Corresponding densitometric analysis of blots in (**A**). (**D**) Intracellular lipid droplets were stained with oil red O working solution in ox-LDL-treated THP-1-derived macrophages after Pim-2 KD. (**E**) Quantification of positive staining for lipid droplets (n=3 per group). (**F**) mRNA expression of inflammatory cytokines, including IL-6, MCP-1, TLR-4 and TNF-α, was determined by quantitative RT-PCR in ox-LDL-treated THP-1-derived macrophages after Pim-2 KD; the values were normalized to the housekeeping gene GAPDH. (**G**) The concentrations of TC, FC, and CE were determined using the TC/FC Quantification Assay (n=3 per group). The data are represented as the means ± SD of three independent experiments; *P < 0.05, **P < 0.01, versus the control group; ^Δ^*P* < 0.05, ^ΔΔ^*P* < 0.01, versus the NC group; ^#^*P* < 0.05 versus the ox-LDL-treated NC group.

Compared with the ox-LDL-treated NC group, the protein expression levels of the mTORC1 signaling pathway and concentrations of intracellular lipid droplets were significantly elevated in ox-LDL-treated THP-1-derived macrophages after the knockdown of Pim-2 (Pim-2 KD) (Figure 3A–3E, respectively). Similarly, compared with the ox-LDL-treated NC group, the concentrations of TC, FC, and CE were also markedly increased in ox-LDL-treated THP-1-derived macrophages after Pim-2 KD (203.5 ±11.2 nmol/mg versus 313.6 ±21.3 nmol/mg (p<0.05), 134.8±20.1 nmol/mg versus 184.8 ±10.1 nmol/mg (p<0.05), 68.7 ±12.3 nmol/mg versus 128.8 ±12.9 nmol/mg (p<0.05), respectively; Figure 3G). Furthermore, compared with the ox-LDL-treated NC group, the mRNA expression of inflammatory cytokines was significantly elevated in ox-LDL-treated THP-1-derived macrophages after Pim-2 KD (Figure 3F).

### Anti-inflammatory role of Pim-2 in atherosclerosis

**Figure 4:**
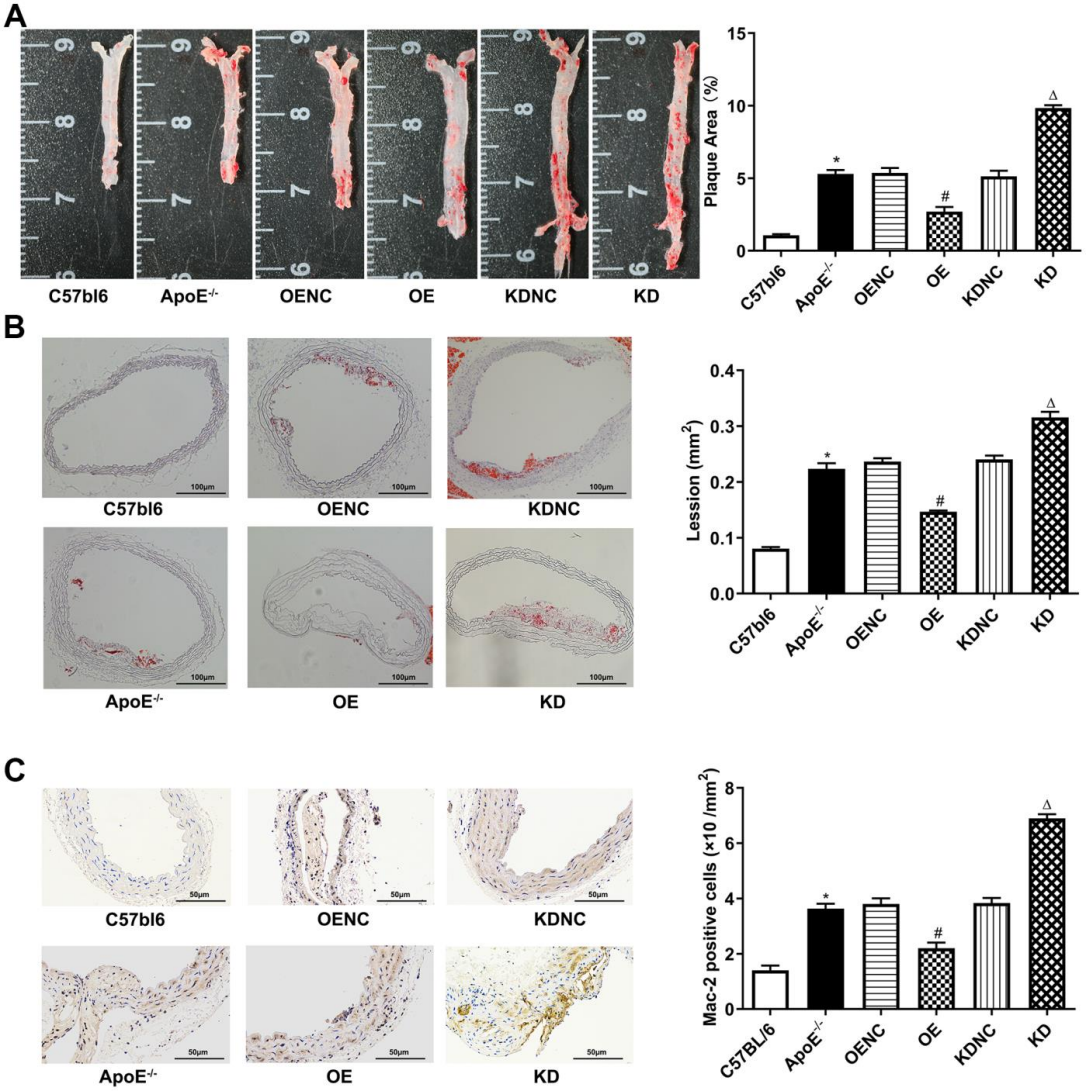
**Antiatherosclerotic role of Pim-2 in atherosclerosis.** Representative images of atherosclerotic lesions stained by oil red O in the whole enfaced aorta (**A**) and aortic root sections (**B**), with semiquantitative analysis on the right (n=4 per group). (**C**) Representative images of Mac-2 expression by immunohistochemistry in mouse aortic root sections, with semiquantitative analysis on the right (n=4 per group). *P < 0.05 versus the C57bl6 group; ^#^*P* < 0.05 versus the OENC group; ^Δ^*P* < 0.05 versus the KDNC group.

**Figure 5:**
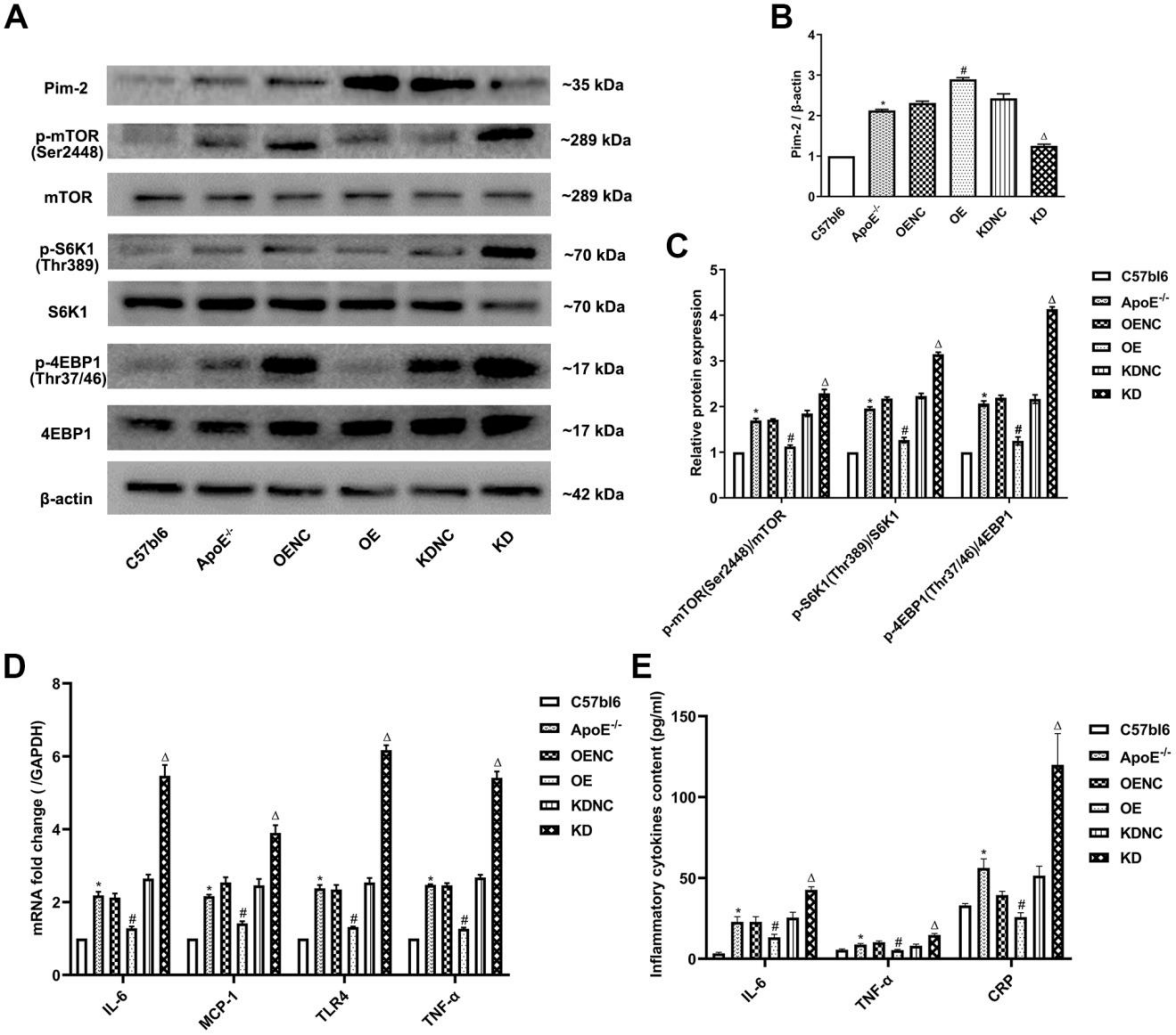
**Anti-inflammatory role of Pim-2 in atherosclerosis via mTOR pathway regulation.** (**A**) Representative western blot analysis of Pim-2, p-mTOR (Ser2448), mTOR, p-S6K1 (Thr389), S6K1, p-4EBP1 (Thr37/46) and 4EBP1 in the aortas of mice. β-Actin was used as a loading control (n=4 per group). (**B**, **C**) Corresponding densitometric analysis of blots in (**A**). (**D**) mRNA expression of inflammatory cytokines, including IL-6, MCP-1, TLR-4 and TNF-α, was determined by quantitative RT-PCR in the aortas of mice (n=4 per group); the values were normalized to the housekeeping gene GAPDH. (**E**) The concentrations of inflammatory cytokines, including IL-6, TNF-α and CRP, detected by ELISA in blood samples of mice (n=6 per group). *P < 0.05 versus the C57bl6 group; ^#^*P* < 0.05 versus the OENC group; ^Δ^*P* < 0.05 versus the KDNC group.

Compared with the C57bl6 wild-type (WT) group, the plaque areas and lesions in the whole aorta and aortic root sections as well as Mac-2 expression by immunohistochemistry were significantly increased in ApoE ^-/-^ control group, but markedly decreased in the OE group and obviously increased in the KD group compared with the NC group (Figure 4A–4C, respectively). Furthermore, compared with the WT group, the protein expression levels of the mTOR signaling pathway were significantly increased in the ApoE ^-/-^ control group, but visibly decreased in the OE group and increased in the KD group compared with those in the NC group (Figure 5A–5C). Furthermore, compared with the WT group, the mRNA expression and concentrations of inflammatory cytokines were significantly elevated in the ApoE ^-/-^ control group, but markedly decreased in the OE group and obviously increased in the KD group compared with those in the NC group (Figure 5D, 5E, respectively).

### mTOR agonist treatment counteracts the anti-inflammatory effect of Pim-2 in THP-1-derived macrophages

**Figure 6:**
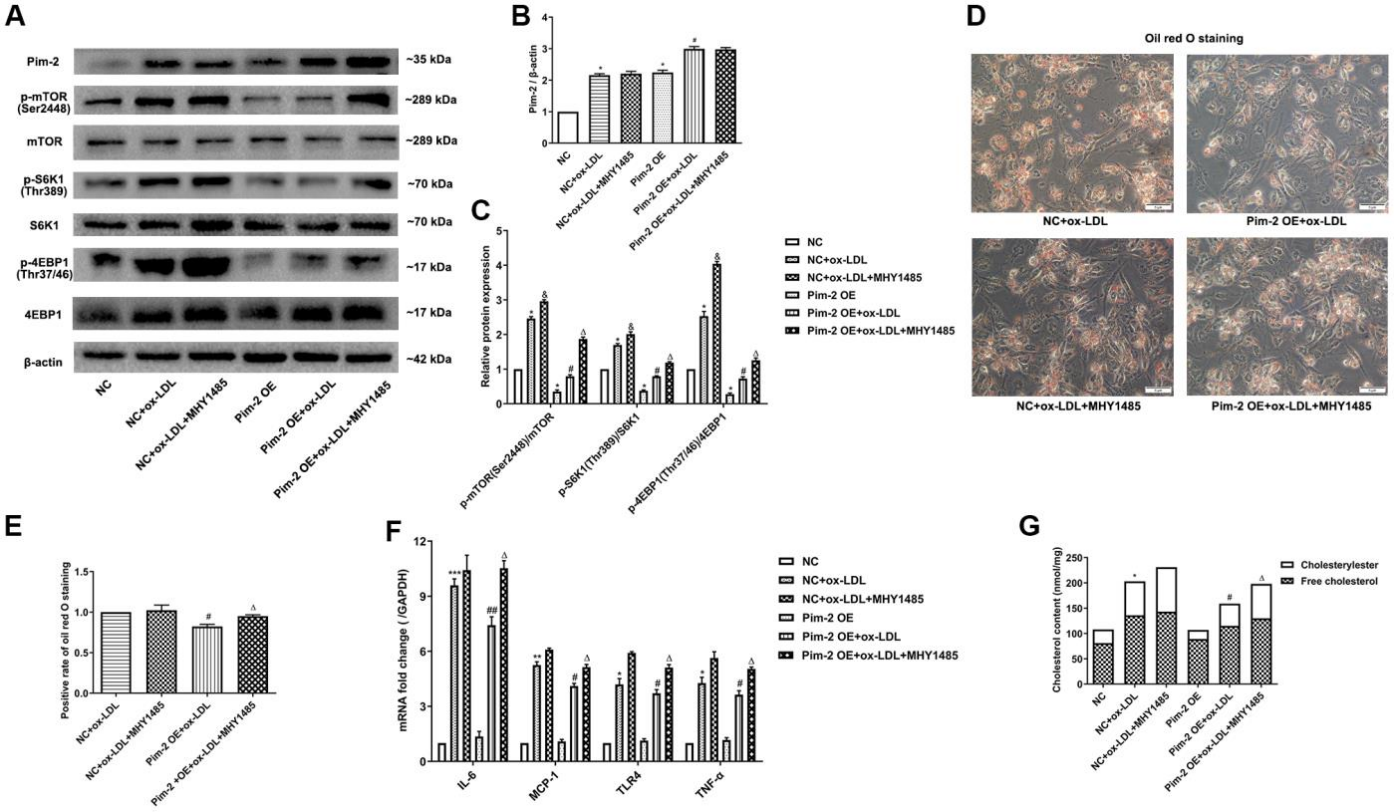
**mTOR agonist treatment counteracts the anti-inflammatory effect of Pim-2 in THP-1-derived macrophages.** (**A**) Representative western blot analysis of Pim-2, p-mTOR (Ser2448) and mTOR, p-S6K1 (Thr389) and S6K1, and p-4EBP1 (Thr37/46) and 4EBP1 in MHY1485-exposed ox-LDL-treated THP-1-derived macrophages after Pim-2 OE. β-Actin was used as a loading control. (**B**, **C**) Corresponding densitometric analysis of blots in (**A**). (**D**) Intracellular lipid droplets were stained with oil red O working solution in MHY1485-disposed ox-LDL-treated THP-1-derived macrophages after Pim-2 OE. (**E**) Quantification of positive staining for lipid droplets (n=3 per group). (**F**) mRNA expression of inflammatory cytokines, including IL-6, MCP-1, TLR-4 and TNF-α, was determined by quantitative RT-PCR in MHY1485-exposed ox-LDL-treated THP-1-derived macrophages after Pim-2 OE; the values were normalized to the housekeeping gene GAPDH. (**G**) The concentrations of TC, FC, and CE were determined using the TC/FC Quantification Assay (n=3 per group). The data are represented as the means ± SD of three independent experiments; *P < 0.05, **P < 0.01, **P < 0.001 versus the NC group; ^&^*P* < 0.05 versus the ox-LDL-treated NC group; ^#^*P* < 0.05, ^##^*P* < 0.01 versus the Pim-2 OE group; ^Δ^*P* < 0.05 versus the Pim-2 OE group.

Compared with the ox-LDL-treated NC group, the protein expression levels of the mTORC1 signaling pathway and mRNA expression of inflammatory cytokines were significantly lowered in ox-LDL-treated THP-1-derived macrophages after Pim-2 OE, but MHY1485 markedly counteracted this downtrend (Figure 6A–6C, 6F, respectively). Likewise, compared with the ox-LDL-treated NC group, the intracellular lipid droplets and concentrations of TC, FC and CE were statistically significantly lowered in ox-LDL- treated THP-1-derived macrophages after Pim-2 OE, but MHY1485 also markedly counteracted this downtrend (Figure 6D, 6E, 6G, respectively).

### mTOR inhibitor treatment rescues the proinflammatory effect of Pim-2 KD in THP-1-derived macrophages

**Figure 7:**
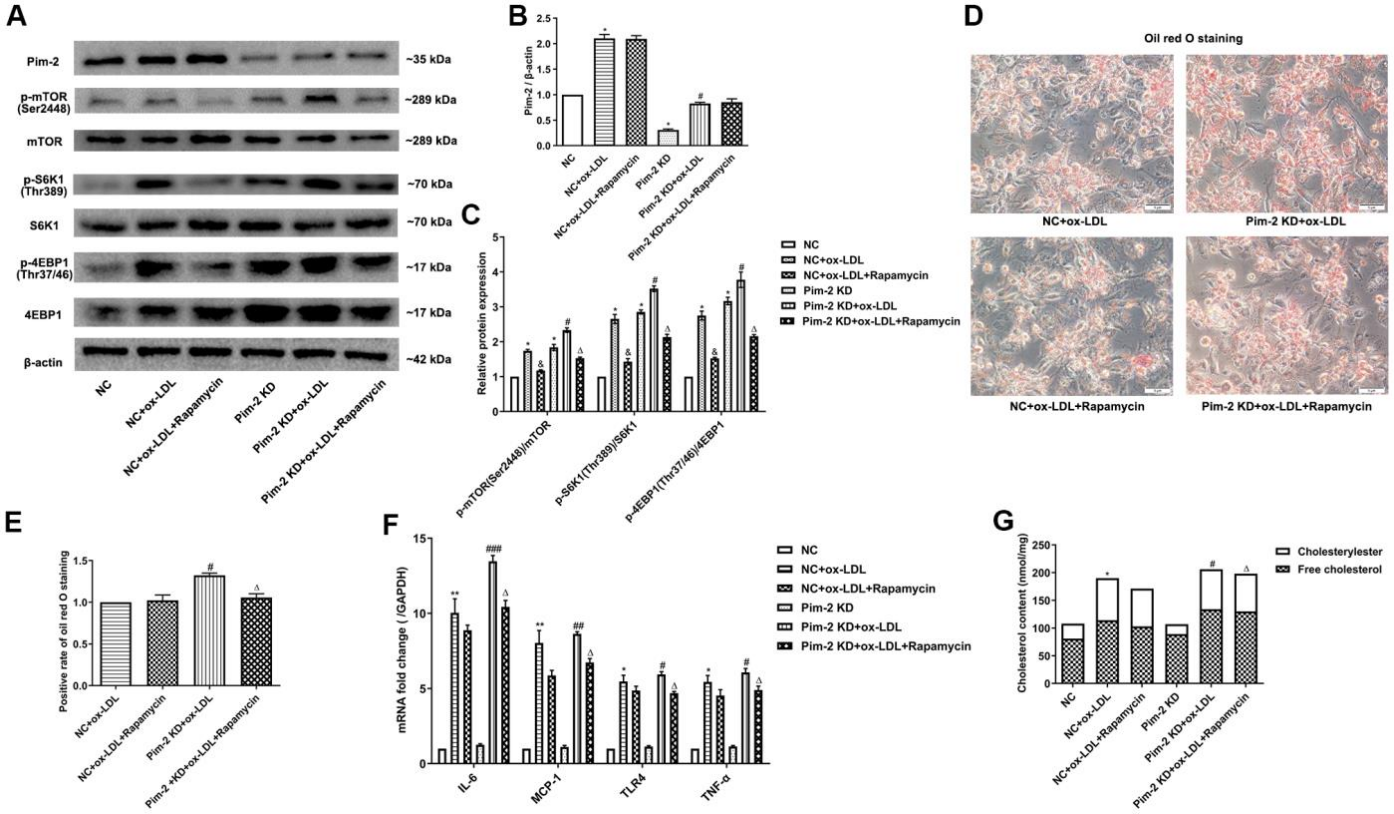
**mTOR inhibitor treatment rescues the proinflammatory effect of Pim-2 KD in THP-1-derived macrophages.** (**A**) Representative western blot analysis of Pim-2, p-mTOR (Ser2448) and mTOR, p-S6K1 (Thr389) and S6K1, and p-4EBP1 (Thr37/46) and 4EBP1 in rapamycin-exposed ox-LDL-treated THP-1-derived macrophages after Pim-2 KD. β-Actin was used as a loading control. (**B**, **C**) Corresponding densitometric analysis of blots in (**A**). (**D**) Intracellular lipid droplets were stained with oil red O working solution in rapamycin-disposed ox-LDL-treated THP-1-derived macrophages after Pim-2 KD. (**E**) Quantification of positive staining for lipid droplets (n=3 per group). (**F**) mRNA expression of inflammatory cytokines, including IL-6, MCP-1, TLR-4 and TNF-α, was determined by quantitative RT-PCR in rapamycin-treated ox-LDL-treated THP-1-derived macrophages after Pim-2 KD; the values were normalized to the housekeeping gene GAPDH. (**G**) The concentrations of TC, FC, and CE were determined using the TC/FC Quantification Assay (n=3 per group). The data are represented as the means ± SD of three independent experiments; *P < 0.05, **P < 0.01 versus the NC group; ^&^*P* < 0.05 versus the ox-LDL-treated NC group; ^#^*P* < 0.05, ^##^*P* < 0.01, ^###^*P* < 0.001 versus the Pim-2 KD group; ^Δ^*P* < 0.05 versus the Pim-2 KD group.

Compared with the ox-LDL-treated NC group, the protein expression levels of the mTORC1 signaling pathway and mRNA expression of inflammatory cytokines were significantly increased in ox-LDL-treated THP-1-derived macrophages after Pim-2 KD, but rapamycin visibly rescued the proinflammatory effect (Figure 7A–7C, 7F, respectively). Likewise, compared with the ox-LDL-treated NC group, the intracellular lipid droplets and concentrations of TC, FC and CE were statistically significantly raised in ox-LDL-treated THP-1-derived macrophages after Pim-2 KD, but Rapamycin also markedly rescued the pro-inflammatory effect (Figure 7D, 7E, 7G, respectively).

### Pim-2 suppresses atherosclerotic inflammation via the mTORC1 pathway in THP-1-derived macrophages

**Figure 8:**
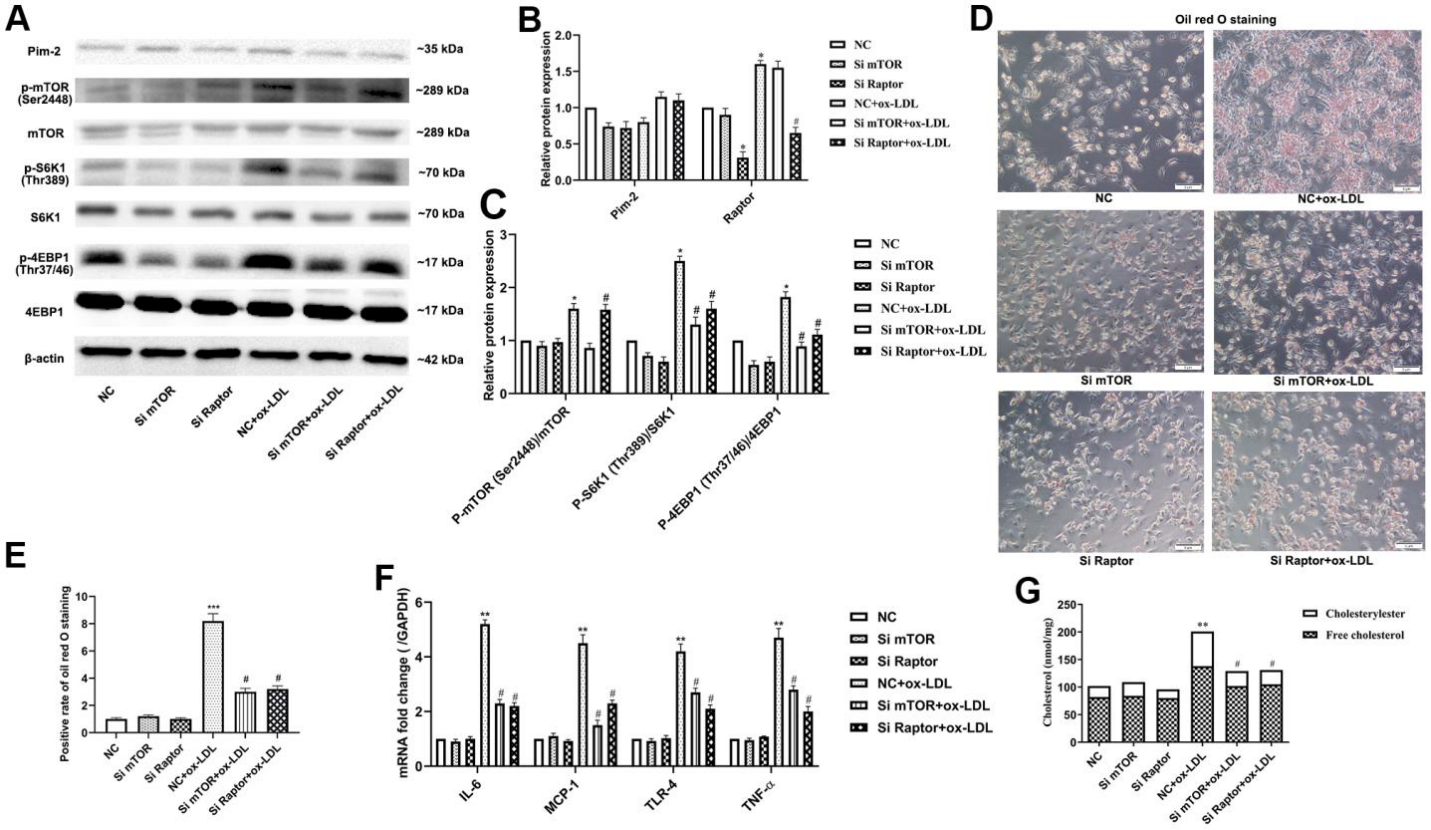
**Pim-2 suppresses atherosclerotic inflammation via the mTORC1 pathway in THP-1-derived macrophages.** (**A**) Representative western blot analysis of Pim-2, p-mTOR (Ser2448) and mTOR, p-S6K1 (Thr389) and S6K1, and p-4EBP1 (Thr37/46) and 4EBP1 in Si-mTOR- or Si-Raptor-treated ox-LDL-treated THP-1-derived macrophages after Pim-2 KD. β-Actin was used as a loading control. (**B**, **C**) Corresponding densitometric analysis of blots in (**A**). (**D**) Intracellular lipid droplets were stained with oil red O working solution in Si-mTOR- or Si-Raptor-disposed ox-LDL-treated THP-1-derived macrophages after Pim-2 KD. (**E**) Quantification of positive staining for lipid droplets (n=3 per group). (**F**) mRNA expression of inflammatory cytokines, including IL-6, MCP-1, TLR-4 and TNF-α, was determined by quantitative RT-PCR in Si-mTOR- or Si-Raptor-treated ox-LDL-treated THP-1-derived macrophages after Pim-2 KD; the values were normalized to the housekeeping gene GAPDH. (**G**) The concentrations of TC, FC, and CE were determined using the TC/FC Quantification Assay (n=3 per group). Data are represented as the means ± SD of three independent experiments; *P < 0.05, **P < 0.01, ***P < 0.001 versus the NC group; ^#^*P* < 0.05 versus the ox-LDL-treated NC group.

Rapamycin is widely used as a complete inhibitor of mTORC1, but several studies have shown that rapamycin also inhibits mTORC2 activity [19, 20]. In our study, the mTORC1 signaling pathway was further significantly activated after Pim-2 KD in ox-LDL-treated THP-1-derived macrophages, but rapamycin markedly rescued the increasing trend (Figure 7A–7C). Therefore, we constructed Si-mTOR and Si-Raptor to explore the underlying mechanics. Compared with the ox-LDL-treated NC group, Si-mTOR and Si-Raptor downregulated the protein expression levels of p-S6K1(Thr389) and p-4EBP1(Thr37/46) and the mRNA expression of inflammatory cytokines in ox-LDL-treated THP-1-derived macrophages in the background of Pim-2 KD (Figure 8A–8C, 8F, respectively). As expected, compared with the ox-LDL-treated NC group, Si-mTOR and Si-Raptor also lowered the intracellular lipid droplets and the concentrations of TC, FC and CE in ox-LDL-treated THP-1-derived macrophages in the background of Pim-2 KD (Figure 8D, 8E, 8G, respectively).

## DISCUSSION

Inflammatory immunity theory has raised considerable concern in the pathogenesis of atherosclerosis [2–6]. We found that the protein expression of Pim-2 is upregulated in ox-LDL-treated THP-1-derived macrophages and an atherosclerotic mouse model, finding that is consistent with clinical phenomenology that Pim-2 is upregulated in atherosclerotic arteries in coronary artery disease patients [21]. Additionally, ox-LDL upregulated the protein expression of p-mTOR, p-S6K1 and p-4EBP1, intracellular lipid droplets, free cholesterol and cholesterylester and the mRNA expression of inflammatory cytokines, including IL-6, MCP-1, TLR-4 and TNF-α, in THP-1-derived macrophages. Functionally, Pim-2 OE obviously attenuated atherosclerotic inflammation associated with the mTORC1 signaling pathway *in vitro* and *in vivo*, whereas Pim-2 KD markedly promoted atherosclerotic inflammation associated with upregulation of the mTORC1 signaling pathway. The plaque areas and lesions in the whole aorta and aortic root sections were alleviated in ApoE ^-/-^ mice with Pim-2 OE, but aggravated by Pim-2 KD. Additionally, an mTOR agonist (MHY1485) counteracted the anti-inflammatory effect of Pim-2 in ox-LDL-treated THP-1-derived macrophages after Pim-2 OE, whereas rapamycin rescued atherosclerotic inflammation in ox-LDL-treated THP-1-derived macrophages after Pim-2 KD. Furthermore, si-mTOR and si-Raptor alleviated the atherosclerotic proinflammatory effect in ox-LDL-treated THP-1-derived macrophages in a background of Pim-2 KD. These results indicated that Pim-2 kinase inhibits atherosclerotic inflammation by suppressing the mTORC1 pathway.

Previous reports [17] have suggested that Pim-2 protein expression is increased in 4-HNE-induced intrinsic inflammation within human rheumatoid arthritis synovial cells. Exogenous Pim-2 overexpression blocked NF-κB activation and the COX-2-mediated inflammatory response by activating the mTORC1 pathway [17]. Additionally,, Pim-2 expression was increased in natural Treg cells from patients with asthma, where a Pim-2 inhibitor attenuated asthma symptoms and improved airway hyperresponsiveness and inflammation compared with asthmatic mice without Pim-2 inhibition [22]. Furthermore, Pim-2 was overexpressed in a subset of human male germ cells and prostate tumors, correlating with the inflammatory response [23]. Multiple studies have also suggested that other members of the Pim kinase family regulate inflammation in many diseases. The inflammatory cytokines IL-6 and IL-27 upregulate the expression of Pim-1 in Sca-1+ cardiac resident stem cells [24, 25]. Pim-1 kinase inhibits the release of the proinflammatory factor IL-1α from airway epithelial cells in allergic asthma caused by house dust mites [26]. Pim-1-deficient mice show strong neutrophil airway inflammation after exposure to cigarette smoke [27]. Pim-3 alleviates lipopolysaccharide-stimulated AR42J pancreatic acinar cell injury by modifying the inflammatory microenvironment [28].

Inflammation is an important pathological mechanism of atherosclerosis [29, 30]. The levels of proinflammatory cytokines, including TNF-α, IL-6, CRP and IL-1, are increased in atherosclerosis patients and animal models, which damage the endothelial surface, recruit and activate various responsive cell populations, and play an inevitable indispensable role in triggering and perpetuating atherosclerosis [31, 32]. Activation of the inflammatory response in atherosclerosis is mainly involved in the Toll-like receptor (TLR), NF-κB, and mTOR-dependent signaling pathways [33–38]. Inhibition of mTOR can defend against the development of atherosclerosis [39]. Yu et al. [40] reported that TLR4 is upregulated by mTOR activation during THP-1-derived macrophage foam cell formation, while rapamycin and Si-mTOR reverse foam cell formation by inhibiting the upregulation of TLR4 expression. Taken together, the inflammatory signaling pathways above may exert a therapeutic effect onatherosclerosis development.

The mTOR signaling pathway contains two different protein multicomplexes [9], of which mTORC1 is necessary to maintain the physiological functions of the heart, and provides protective effects under cardiac stress conditions [39, 41]. Numerous reports have found that mTORC1 inhibition limits the progression of atherosclerosis by improving endothelial function, inhibiting smooth muscle cell proliferation, decreasing macrophage content, minimalizing monocyte recruitment, and decreasing lipid accumulation [33, 42]. Additionally, Pim-2 regulates cell growth and survival by activating the mTORC1 signaling pathway [17, 43]. Rapamycin is widely used as a complete inhibitor of mTORC1 by interacting with the intracellular FK506-binding protein, but several studies have shown that rapamycin also inhibits mTORC2 activity [19, 20]. In our study, we constructed Si-mTOR and Si-Raptor to explore the underlying mechanics. We found that si-mTOR and si-Raptor alleviated the atherosclerotic proinflammatory effect in ox-LDL-treated THP-1-derived macrophages in a the background of Pim-2 KD.

This study has some limitations. Our current data cannot exclude the role of Pim-2 in other cell types, such as vessel endothelial cells and smooth muscle cells in atherosclerosis progression. Additionally, we did not observe the location distribution of Pim-2 in the aorta, information that might be useful for further preclinical and clinical studies. Future studies should be performed using cell-specific Pim-2-knockout mice.

In conclusion, these results indicated that Pim-2 kinase inhibits atherosclerotic inflammation by suppressing the mTORC1 pathway. Our findings offer novel insight that Pim-2 may serve as a therapeutic target in atherosclerosis.

## MATERIALS AND METHODS

### Cell culture and transfections

THP-1 monocytes were obtained from the Cell Bank of Shanghai Chinese Academy of Sciences (SCSP-567, Shanghai, China). THP-1 cells were cultured in Roswell Park Memorial Institute medium (RPMI 1640, Invitrogen) supplemented with 10% FBS (Gibco, USA) and 50 pM β-mercaptoethanol (M3148, Sigma, USA) in a humidified atmosphere of 5% CO2 at 37° C and grown to 70–80% confluence. Cells at passages three to eight were used in the experiments. THP-1 monocytes are differentiated into macrophages by 48 h incubation with 150 nM phorbol 12-myristate 13-acetate (PMA, P1585, Sigma, USA) in RPMI medium. After 48 h treatment with PMA, THP-1 cells became adherent. The PMA-containing medium was removed and the differentiated cells were cultured in fresh cell culture medium for 48 h to increase their cytoplasmic volume. Copper (Cu^2+^) oxidized human low density lipoprotein (ox-LDL) was purchased from the Peking Union-Biololgy (Beijing, China). THP-1-derived macrophages were sequentially treated with 50ug/ml ox-LDL for 12h, 24h, 36h, and 48h, respectively.

### Analysis of CD11b plasma membrane expression by flow cytometry

After being washed with phosphate buffered saline (PBS), adherent THP-1-derived macrophages were detached with 5 mM EDTA and placed in FACS tubes. The cell suspension was centrifuged for 5 min at 200 g, and the pellet was resuspended in PBS containing 2% FBS. The pellet was next resuspended in human truStain FcX (#422301; BioLegend) diluted 20 times in PBS. The suspension was then incubated at room temperature for 10 min. Next, the cells were centrifuged for 5 min at 200 g, and primary PE (phycoerythrin) mouse anti-human CD11b antibody (#555388, BD Pharmingen) was used to stain the differentiated cells in PBS in the dark at RT for 30 min. The stained cells were then washed twice with PBS and analyzed on a BD AccuriC6 Plus flow cytometer. Cells were also incubated with PE mouse IgG1, κ isotype control (#555749; BD Pharmingen) corresponding to each primary antibody. Unstained samples were prepared for cell size and granularity assessments. The data were collected and analyzed using FACSCalibur (BD Biosciences, Supplementary Figure 1).

### Recombinant lentivirus and infection of THP-1 cells

The recombinant lentivirus vectors containing Pim-2 sequence and shRNA of Pim-2 was constructed and synthesized by GeneChem (Shanghai, China). The full-length cDNA sequence of human Pim-2 (GenBank no. NM_ 006875) and negative control were synthesized and subcloned into the lentiviral Ubi-MCS-3FLAG-CBh-gcGFP-IRES-puromycin vector (Addgene plasmid GV492). A shRNA sequence targeting human Pim-2 (5′- ccggGATGAACCCTACACTGACTTTctcgagAAAGTCAGTGTAGGGTTCATCtttttg-3′) corresponding to coding regions 571–1591 relative to the first nucleotide of the start codon of human Pim-2 (GenBank no. NM_ 006875) and a scrambled (5′-ccggTTCTCCGAACGTGTCACGTctcgagACGTGACACGTTCGGAGAAtttttg-3′) sequence were cloned into the lentiviral U6-MCS-Ubiquitin-Cherry-IRES-puromycin vector (Addgene plasmid GV298). Lentiviruses were generated by transiently transfecting the resulting lentiviral vectors along with packaging vectors pGC-LV, pHelper 1.0, and pHelper 2.0 into HEK 293T cells (from the human embryonic kidney cell line) using Lipofectamine 2000. The medium containing viruses was harvested 48 h following the start of transfection and concentrated using Millipore Centricon Plus-20 filters.

For lentivirus infection, viral solutions (MOI = 50) were added to cell culture medium containing polybrene (5 μg/ml, H8761, Solarbio, Beijing, China). After 8 hours of infection, fresh RPIM1640 medium was replaced. 48 hours after infection, THP-1 cells were selected using puromycin (1.5 μg/ml, P8230, Solarbio, Beijing, China). The efficiency of lentiviral transfection was determined with fluorescence microscopy, PCR and western blot analysis (Supplementary Figures 2, 3). The transfected cells were then treated with PMA for 48h to induce into macrophages for the subsequent studies.

### Cholesterol/cholesteryl ester quantification

The concentrations of total cholesterol (TC), free cholesterol (FC) and cholesteryl ester (CE) were determined using the TC/FC Quantification Assay (E1015, E1016, Applygen, Beijing, China) according to the manufacturer's protocol. The fluorometric assay was performed using a microplate reader (Bio-Rad, USA) at an absorption wavelength of 550 nm. The TC and FC results were presented as nmol/mg, and CE was determined by subtracting the value of FC from TC.

### mTOR inhibitor and agonist

The mTOR inhibitor rapamycin (A8167) and agonist MHY1485 (B5853) were purchased from APExBIO (Shanghai, China). THP-1-derived macrophages were treated with 100 nM rapamycin for 1 h or 20 μM MHY1485 for 30 min to inhibit or activate the mTOR pathway before exposure to 50 μg/ml of ox-LDL for 24 h.

### Small interfering RNA (siRNA) transfection

Gene expression silencing was achieved using siRNA-specific target sequences. Human mTOR siRNA (Si-mTOR) and Raptor siRNA (Si-Raptor) were obtained from Gene Pharma (Shanghai, China). The sense and antisense strands of siRNA are presented in Supplementary Table 1. After the lentivirus transfected cells (knockdown of Pim-2) were treated with PMA for 48 h to induce macrophages in six-well plates (1.5*10^5^ cell per well), this THP-1-derived macrophages were then transiently transfected with 100 nM siRNA target sequences or negative control siRNA using GP-transfect-Mate (G04009; GenePharma, Shanghai, China) according to the manufacturer’s protocol. The transfected cells were then exposed to 50 μg/ml of ox-LDL for 24 h for subsequent studies.

### Animal studies

Male 7-week-old C57BL/6 mice (n=12) and ApoE ^-/-^ mice (n=60) were obtained from the Department of Laboratory Animal Science of Peking University Health Science Center (Beijing, China) and were housed conventionally at 22± 2.0° C and 50% ± 5% humidity under a 12-h light-dark cycle with free access to water and food. All the animal experiments were performed and analyzed by blinded experimenters. All the animal experiments were conducted in compliance with the National Institutes of Health (NIH) policies in the Guide for the Care and Use of Laboratory Animals and were approved by the Nanchang University Animal Care and Use Committee (SYXK(G) 2015-0001).

After one week of adaptive feeding, all the mice were fed a high-fat diet (21% fat and 0.3% cholesterol, MD12015, Medicinece, Jiangsu, China) for 12 weeks to create an atherosclerotic model [13]. Body weights were monitored once every 2 weeks, and the levels of fasting blood glucose and blood lipids were tested both at baseline and at 20 weeks of age (Supplementary Figure 4). At 8 weeks of age, the ApoE ^-/-^ mice were assigned to five groups randomly : (i) ApoE ^-/-^ control group that received a single tail vein injection of equivoluminal PBS at 12 weeks of age (n = 12); (ii) Overexpression-negative control group (OENC, n = 12) that received a single tail vein injection of negative control virus corresponding to Pim2-overexpressing virus at 12 weeks of age; (iii) Pim2-overexpressing group (OE, n = 12) that received a single tail vein injection of Pim2-overexpressing virus at 12 weeks of age; (iv) Knockdown -negative control group (KDNC, n = 12) that received a single tail vein injection of negative control virus corresponding to Pim2-knockdown virus at 12 weeks of age; (v) Pim2-knockdown (KD, n = 12) that received a single tail vein injection of Pim2-knockdown virus at 12 weeks of age.

Eight weeks after the tail vein injection, the mice were anesthetized using an i.p. injection of sodium pentobarbital (50 mg/kg), and the adequacy of anesthesia was confirmed by the absence of a reflex response to a foot squeeze. Afterward, blood samples were collected under anesthesia by cardiac puncture immediately before sacrifice. Next, the hearts were perfused and the aortas were harvested. Partial aortas were fixed in 4% paraformaldehyde for pathological analysis. The remaining aortas were freshly flash-frozen in liquid nitrogen for gene and protein expression analysis.

### Recombinant adeno-associated virus (rAAV) and infection of mice

The AAV2/8-CMV-NC recombinant, AAV2/8-CMV-Pim2 recombinant, AAV2/8-U6-NC recombinant and AAV2/8-U6-shPim-2 recombinant were constructed and ordered from Genechem (Shanghai, China), and the titers were ~2 × 10^12^ viral genomes per ml (vg/ml). To produce vessel-specific rAAV, mouse Pim2 genes (GenBank no. NM_138606) was sub-cloned into the CMV-bGlobin-MCS-EGFP-3FLAG-WPRE-hGH-polyA vector (Addgene plasmid GV388). A shRNA sequence targeting mouse Pim-2 (5′- accggGGAAGGTGGGTGAAGGCAAttcaagagaTTGCCTTCACCCACCTTCCttttt-3′) corresponding to coding regions 440–458 relative to the first nucleotide of the start codon of human Pim-2 (GenBank no. NM_138606) and a scrambled (5′- accgg CGCTGAGTACTTCGAAATGTCttcaagaga GACATTTCGAAGTACTCAGCGttttt-3′) sequence were cloned into the U6-MCS-CAG-mCherry vector (Addgene plasmid GV480). rAAV were generated by transiently transfecting the resulting vectors along with packaging vectors pAAV-RC and Paav Helper into AAV 293T cells using CaCl_2_. The medium containing viruses was harvested 48 h following the start of transfection and concentrated using Millipore Centricon Plus-20 filters. Viral particles were purified by an iodixanol step-gradient ultracentrifugation method. The titers were ~2 × 10^12^ viral genomes per ml (vg/ml).

In the AAV2/8-overexpressing atherosclerosis model, each mouse from the OENC or OE group received a single tail vein injection of 3 ×10^11^ genome copies of AAV2/8-CMV-NC recombinant or AAV2/8-CMV-Pim2 recombinant at 12-week-old. In the AAV2/8-knockdown atherosclerosis model, each mouse from the KDNC or KD group received a single tail vein injection of 3 ×10^11^ genome copies of AAV2/8-U6-NC recombinant or AAV2/8-U6-shPim-2 recombinant at 12-week-old.

### Oil red O staining

The oil red O working solution was prepared by diluting the stock solution with distilled water (3:2). For staining, THP-1-derived macrophages were fixed in 10% paraformaldehyde. After being washed with PBS and dehydrated with 60% isopropanol, oil red O working solution was added to the culture dishes and incubated at 37° C for 10 min. Next, the cells were washed with 60% isopropanol until the intercellular space was clear using an optical microscope (Olympus, Tokyo, Japan).

At the end of the administration, the aortas were harvested, the adventitial fat was carefully removed, the whole aortas were opened longitudinally, and aortic root sections were cut to an 8-μm thickness. The aortas were stained with oil red O working solution as indicated above and then differentiated with 50% glycerol. The intracellular lipid droplets and lesion areas were quantified using Image-Pro Plus 6.0 (Media Cybernetics, USA).

### Immunohistochemistry

Immunohistochemistry was performed to detect the macrophage marker protein Mac-2. Briefly, the aortas were embedded in paraffin, and 8-μm slices were cut from the embedded blocks. Rehydrated sections were incubated with Mac-2 antibodies (GB12246, Servicebio, Wuhan, China, 1:500). The cell nuclei were stained with 4′,6-diamidino-2-phenylindole (DAPI, C0065, Solarbio, Beijing) and counterstained with hematoxylin (G1080, Solarbio, Beijing). Semiquantitative analysis of the tissue staining was performed using Image-Pro Plus 6.0 (Media Cybernetics, USA).

### Enzyme linked immunosorbent assay (ELISA)

The concentrations of inflammatory cytokines including tumor necrosis factor-α (TNF-α), interleukin-6 (IL-6) and C reactive protein (CRP) were quantified using the ELISA kits (#88-7324-88, #88-7064-88, Invitrogen, USA; and #SEA821, USCN KIT INC, Wuhan, China) according to the manuscript’s instructions. Briefly, serum samples of mice were added into 96-well plates coated with anti-TNF-α, anti-IL-6, and anti-CRP antibodies after blocking with 1% bovine serum albumin, followed by incubation with tetramethylbenzidine (TMB, T8120, Solarbio, Beijing, China), and the reaction was stopped by 50μl of Stop Solution.

### Quantitative real-time PCR

Total RNA was extracted from the aortas and cells using TRIzol reagent (Invitrogen Life Technologies, Carlsbad, CA) according to the manufacturer's protocol. cDNA synthesis was performed using a TaqMan reverse transcription kit (KR118-03, Tiangen, Beijing, China). Then, quantitative real-time PCR was performed using a SuperReal PreMix Plus (SYBR Green) Kit (FP205, Tiangen, Beijing, China) on a 7500 Fast Real Time PCR system from Applied Biosystems (Bio-Rad, USA). All RT-PCR analysis was performed in duplicate 20-μl reactions in 96-well plates. The resulting melting curves were visually inspected to ensure specificity of product detection. Primer sequences were listed in Supplementary Table 2. The relative gene expression levels were determined by the 2^–ΔΔCT^ using GAPDH as a reference gene.

### Western blot analysis

The aortas and THP-1-derived macrophages were homogenized and lysed using radioimmunoprecipitation (RIPA) buffer (R0010, Solarbio, Beijing, China) supplemented with cocktail proteinase and phosphatase inhibitors (P6730 and P1260, Solarbio, Beijing, China). Protein concentration was measured with the BCA Protein Assay Kit (PA115, Tiangen, Beijing, China). Equal amounts of protein were separated on SDS-PAGE electrophoresis and electrotransferred onto PVDF membranes (Millipore, USA). The membranes were blocked in TBST buffer and then further incubated primary antibodies (Supplementary Table 3) overnight at -4° C. Then, immunoreactive bands were detected through incubating with secondary antibody (Boster, Wuhan, China) conjugated with horseradish peroxidase (HRP) and visualized using enhanced chemiluminescence reagents (ECL, Thermo Fisher Scientific). In our research, β-actin was used as an internal control.

### Statistical analysis

All data were presented as means ± standard deviation (SD) using the Graphpad Prism 8.0 software (GraphPad Software Inc., CA, USA). Normality analysis was performed before any testing, via the Shapiro-Wilk test. Differences were evaluated using unpaired Student’s t test between two groups. One-way ANOVA was conducted followed by Bonferroni post hoc test for comparisons between >2 groups. The non-normal distributed data were analyzed using non-parametric testing (Mann-Whitney U test for two groups and Krushal-Wallis H test for >2 groups). Differences between groups over time were evaluated using repeated measures analysis of variance. Statistical analysis was performed using SPSS23.0 (SPSS Inc., Chicago, IL, USA) software and P < 0.05 was considered statistically significant.

### Ethics statement

All animal experiments were conducted in compliance with the National Institutes of Health (NIH) policies in the Guide for the Care and Use of Laboratory Animals and were approved by the Nanchang University Animal Care and Use Committee (SYXK(G) 2015-0001).

### Data availability

The datasets used and/or analyzed during the present study are available from the corresponding author on reasonable request.

## Supplementary Material

Supplementary Figures

Supplementary Tables
