# Supplementary material for: Pim-2 kinase inhibits inflammation by suppressing the mTORC1 pathway in atherosclerosis
Source: Aging (Albany NY). 2021 Sep 21;13(18):22412–31. doi: 10.18632/aging.203547 (PMC8507271; doi:10.18632/aging.203547)
Supplement: Supplementary Figures [file aging-13-203547-s001.pdf]

SUPPLEMENTARY FIGURES

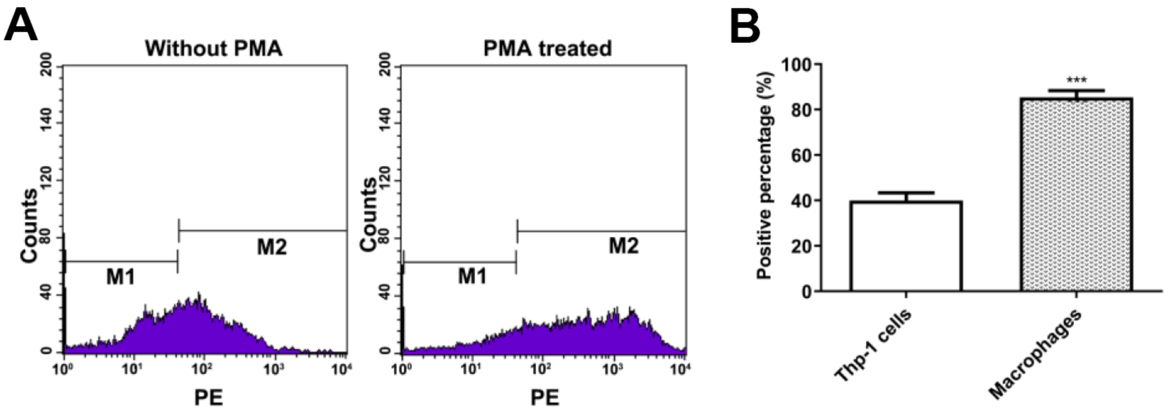

Supplementary Figure 1. Analysis of CD11b plasma membrane expression by flow cytometry in THP-1-derived macrophages. (**A**) Representative image of CD11b plasma membrane expression by flow cytometry in THP-1 cells or THP-1-derived macrophages. (**B**) Quantification analysis of CD11b plasma membrane expression (n=3 per group). Data are represented as the means ± SD of three independent experiments; ****P* < 0.001 versus THP-1 cells group.

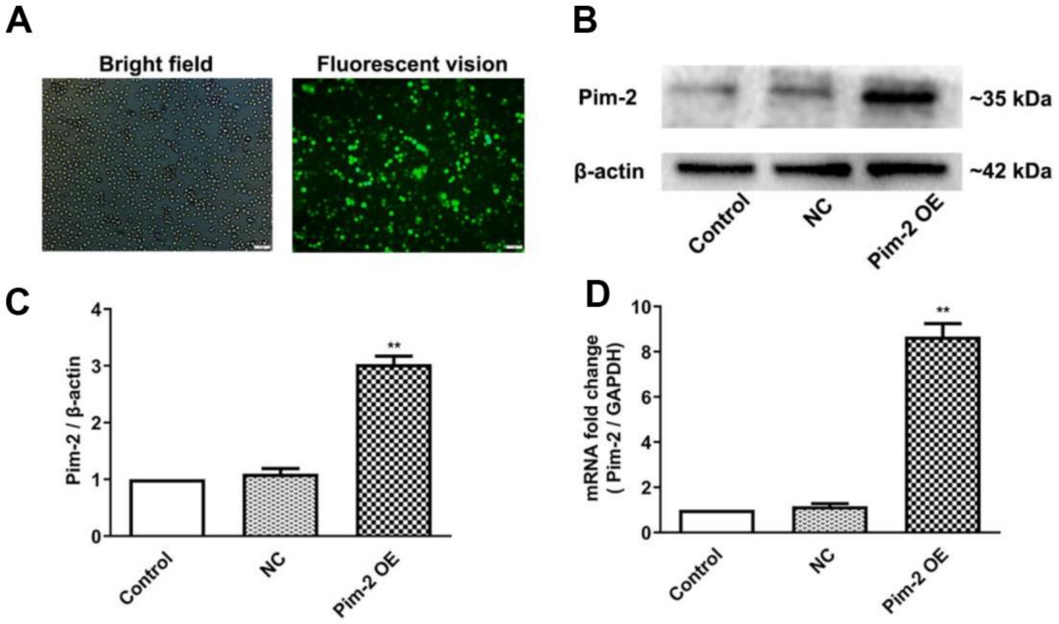

Supplementary Figure 2. The efficiency of lentiviral transfection in THP-1 cells with Pim-2 OE. (**A**) Representative image of lentiviral transfection in THP-1 cells with Pim-2 OE. (**B**) Representative western blot analysis of Pim-2 in THP-1 cells with Pim-2 OE. (**C**) Corresponding densitometric analysis of blots in (**B**, **D**) mRNA expression of Pim-2 by quantitative RT-PCR in THP-1 cells with Pim-2 OE; values normalized to the house-keeping gene, GAPDH. Data are represented as the means ± SD of three independent experiments; ***P* < 0.01 versus NC group.

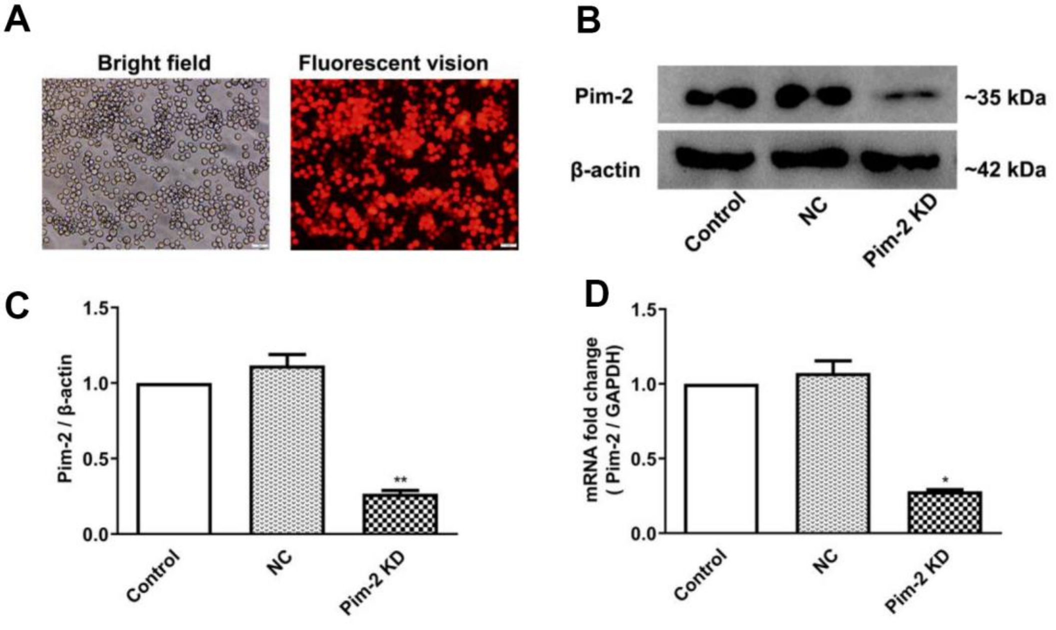

Supplementary Figure 3. The efficiency of lentiviral transfection in THP-1 cells with Pim-2 KD0. (**A**) Representative image of lentiviral transfection in THP-1 cells with Pim-2 KD. (**B**) Representative western blot analysis of Pim-2 in THP-1 cells with Pim-2 KD. (**C**) Corresponding densitometric analysis of blots in (**B**, **D**) mRNA expression of Pim-2 by quantitative RT-PCR in THP-1 cells with Pim-2 KD; values normalized to the house-keeping gene, GAPDH. Data are represented as the means ± SD of three independent experiments; **P* < 0.05, ***P* < 0.01 versus NC group.

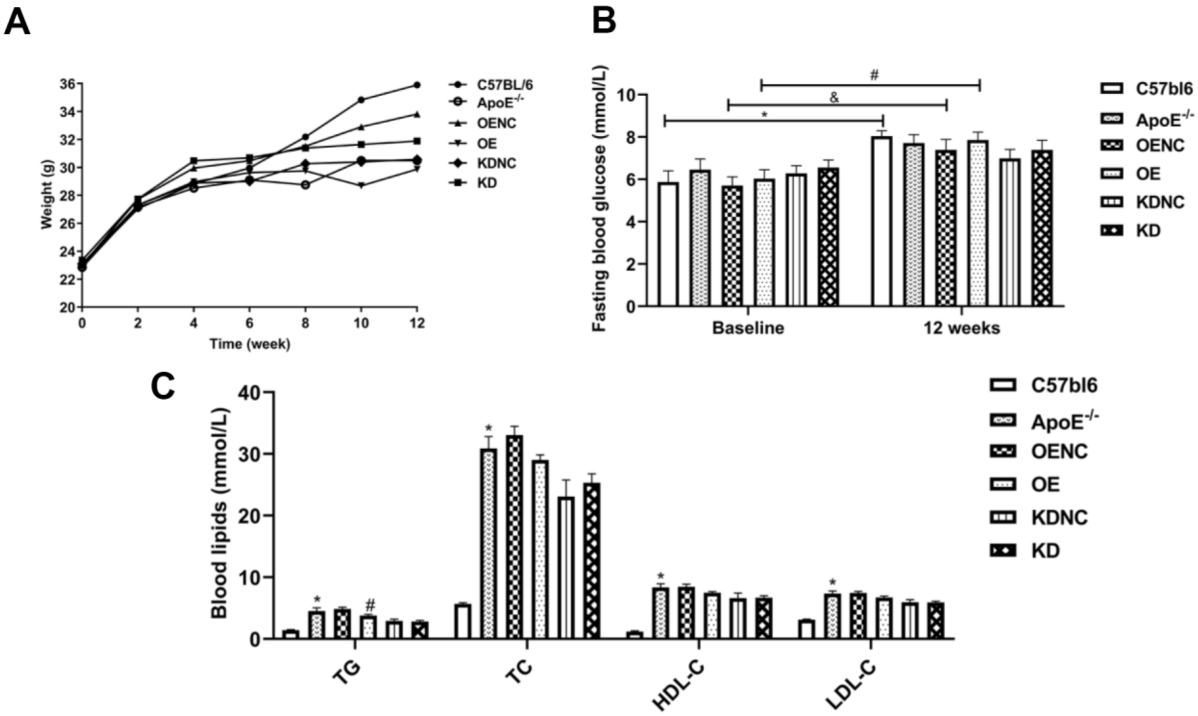

Supplementary Figure 4. The body weight, fasting blood glucose and blood lipids of mice. (**A**) Body weight (g) of mice was recorded at the indicated times (n=12 per group). (**B**) Fasting blood glucose (mmol/L) was determined at baseline and 20-week-old (n=10 per group); **P* < 0.05, $^{\&}P$ < 0.05, $^{\#}P$ < 0.05 versus the corresponding baseline levels of fasting blood glucose. (**C**) The levels of blood lipids at 20-week-old (n=10 per group); **P* < 0.05 versus C57bl6 group; $^{\#}P$ < 0.05 versus OENC group.
