# Supplementary material for: Pim-2 kinase inhibits inflammation by suppressing the mTORC1 pathway in atherosclerosis
Source: Aging (Albany NY). 2021 Sep 21;13(18):22412–31. doi: 10.18632/aging.203547 (PMC8507271; doi:10.18632/aging.203547)
Supplement: Supplementary Tables [file aging-13-203547-s002.pdf]

SUPPLEMENTARY TABLES

Supplementary Table 1. SiRNA sequence.

Name	Sequences (5'→3')
h-Si-Negative control	sense: UUCUCCGAACGUGUCACGUTT<br>antisense: ACGUGACACGUUCGGAGAATT
h-Si-mTOR	sense: CCAAGAUACCAUGAACCAUTT<br>antisense: AUGGUUCAUGGUAUCUUGGTT
h-Si-Raptor	sense: CCCUCAUCGGAGUUUCCUUTT<br>antisense: AAGGAAACUCCGAUGAGGGTT

Supplementary Table 2. The RT-qPCR sequences used in the study.

Gene name	Primer sequences (5'→3')
h-Pim-2	F: CGAAGTCGCACTGCTATGGA<br>R: CTGGARGGCTGCCACTACTT
h-IL-6	F: TGAGGAGACTTGCCTGGTGA<br>R: TGCAGGAACTGGATCAGGAC
h-MCP-1	F: CTTCATTCCCCAAGGGCTCG<br>R: GCTTCTTTGGGACACTTGCTG
h-TLR4	F: TGTGGCTCACAATCTTATCCA<br>R: CTAAATGTTGCCATCCGAAA
h-TNF-α	F: TGGCGTGGAGCTGAGAGATA<br>R: GGAGGTTGACCTTGGTCTGG
h-GAPDH	F: CTGCACCACCAACTGCTTAG<br>R: AGGTCCACCACTGACACGTT
m-IL-6	F: AGCCAGAGTCCTTCAGAGAGA<br>R: GCCACTCCTTCTGTGACTCC
m-MCP-1	F: GATGCAGTTAACGCCCCACT<br>R: CCCATTCCTTCTTGGGGTCA
m-TLR4	F: TCTGGGGAGGCACATCTTCT<br>R: AGGTCCAAGTTGCCGTTTCT
m-TNF-α	F: ATGGCCTCCCTCTCATCAGT<br>R: TTTGCTACGACGTGGGCTAC
m-GAPDH	F: TTGCAGTGGCAAAGTGGAGA<br>R: GGTCTCGCTCCTGGAAGATG

Supplementary Table 3. List of primary antibodies.

Name	Catalog	Analysis	Dilutions	Company
Pim-2	#ab129057	Western blot	1:1000	Abcam
Phospho-mTOR(Ser2448)	#ab109268	Western blot	1:1000	Abcam
mTOR	#ab32028	Western blot	1:1000	Abcam
Phospho-S6K1(Thr389)	#9206	Western blot	1:1000	CST
S6K1	#2708	Western blot	1:1000	CST
Phospho-4EBP1(Thr37/46)	#2855	Western blot	1:1000	CST
4EBP1	#9644	Western blot	1:1000	CST
Raptor	#2280	Western blot	1:1000	CST
JNK1	#ab199380	Western blot	1:1000	Abcam
CD11b	#ab52478	Flow Cyt	1:30	Abcam
β-Actin	# 66009-1-Ig	Western blot	1:5000	Proteintech
